# A multi-centre, randomised trial for diagnostic efficacy of the automatic breast volume scanner ultrasound for breast cancer screening in China

**DOI:** 10.3389/fonc.2024.1421425

**Published:** 2025-01-21

**Authors:** Ying Xu, Yali Xu, Songjie Shen, Feng Mao, Xiaohui Zhang, Yanna Zhang, Yan Lin, Yidong Zhou, Qiang Sun

**Affiliations:** Department of Breast Surgery, Peking Union Medical College Hospital, Chinese Academy of Medical Sciences and Peking Union Medical College, Beijing, China

**Keywords:** breast cancer, breast screening, ABUS, HHUS, ultrasound

## Abstract

**Introduction:**

The US plays a crucial role in screening Asian women for breast disease. ABUS offers several advantages over traditional HHUS, including quicker examination, objectivity, and the ability to store and reconstruct images. This study marks the first large-scale opportunistic screening of ABUS in the population.

**Methods:**

Between January 1, 2016, and December 31, 2019, 10,537 women aged 35–75 years from nine districts were randomly assigned to either HHUS or ABUS groups. Diagnostic methods were quantified, and comparisons were made using the Chi-square test.

**Results:**

The screening groups consisted of 5,445 participants for HHUS and 4,936 for ABUS. The HHUS and ABUS groups identified 90 carcinomas and 292 benign lesions or 71 carcinomas and 178 benign lesions, respectively. SE), SP, AC, PPV, and NPV for HHUS were 51.11%, 93.84%, 93.13%, 12.23%, and 99.13%, respectively, while for ABUS, they were 66.20%, 93.77%, 93.38%, 13.43%, and 98.98%. The area under the curve (AUC) values for HHUS and ABUS were 0.72 (95% CI: 0.67–0.78) and 0.86 (95% CI: 0.82–0.91), respectively, indicating superior diagnostic performance of ABUS over HHUS (Delong test p < 0.05).

**Discussion:**

ABUS is user-friendly, requires minimal training, reduces reliance on examiner experience, and demonstrates potential for superior sensitivity compared to HHUS in breast cancer screening.

## Introduction

Breast cancer is currently the most prevalent cancer among women and a leading cause of cancer-related deaths. In 2020, there were 2.3 million new cases of breast cancer and 685,000 deaths globally (1). China is experiencing a shift towards cancer patterns seen in developed nations, marked by elevated incidences of lung, colorectal, breast, and prostate cancers. According to GLOBOCAN 2020 data, breast cancer is the most commonly diagnosed cancer in females (2). Numerous evidence-based medical studies indicate that early detection and treatment through screening can lower mortality rates and reduce the intensity of required treatment (3). X-ray mammography (MG) is a primary imaging tool for early breast cancer detection in Western countries and is widely utilized for breast cancer screening. However, in dense breast tissue, MG’s diagnostic sensitivity decreases by about 50% (4). Asian women, particularly those under 40 years old, often have smaller and denser breasts compared to Western women. Since MG is less effective for dense breasts, US plays a crucial role in screening for breast disease among Asian women (5).

Ultrasound is non-radioactive and adept at distinguishing between fat and gland tissue echoes, as well as characterizing lesion morphology and boundaries (6). Previous studies have shown that in large-scale screenings, US and MG demonstrate comparable efficiency, with US exhibiting higher sensitivity and specificity in screening dense breasts (7–9). However, conventional ultrasound examinations using handheld probes may result in local omissions due to size limitations and inspector experience. The repeatability and standardization of traditional hand-held ultrasound (HHUS) need enhancement. Introduced in 2009, the automatic breast volume scanner ultrasound (ABUS) offers advantages such as reduced time consumption, objectivity, and consistent retention of inspection data and plane graphics, in contrast to HHUS (10). Hence, ABUS may serve as a valuable tool for breast cancer screening and diagnosis. This study represents the first large-scale population opportunistic screening employing ABUS and compares its diagnostic performance with HHUS.

## Methods

### Study design

**Figure 1:**
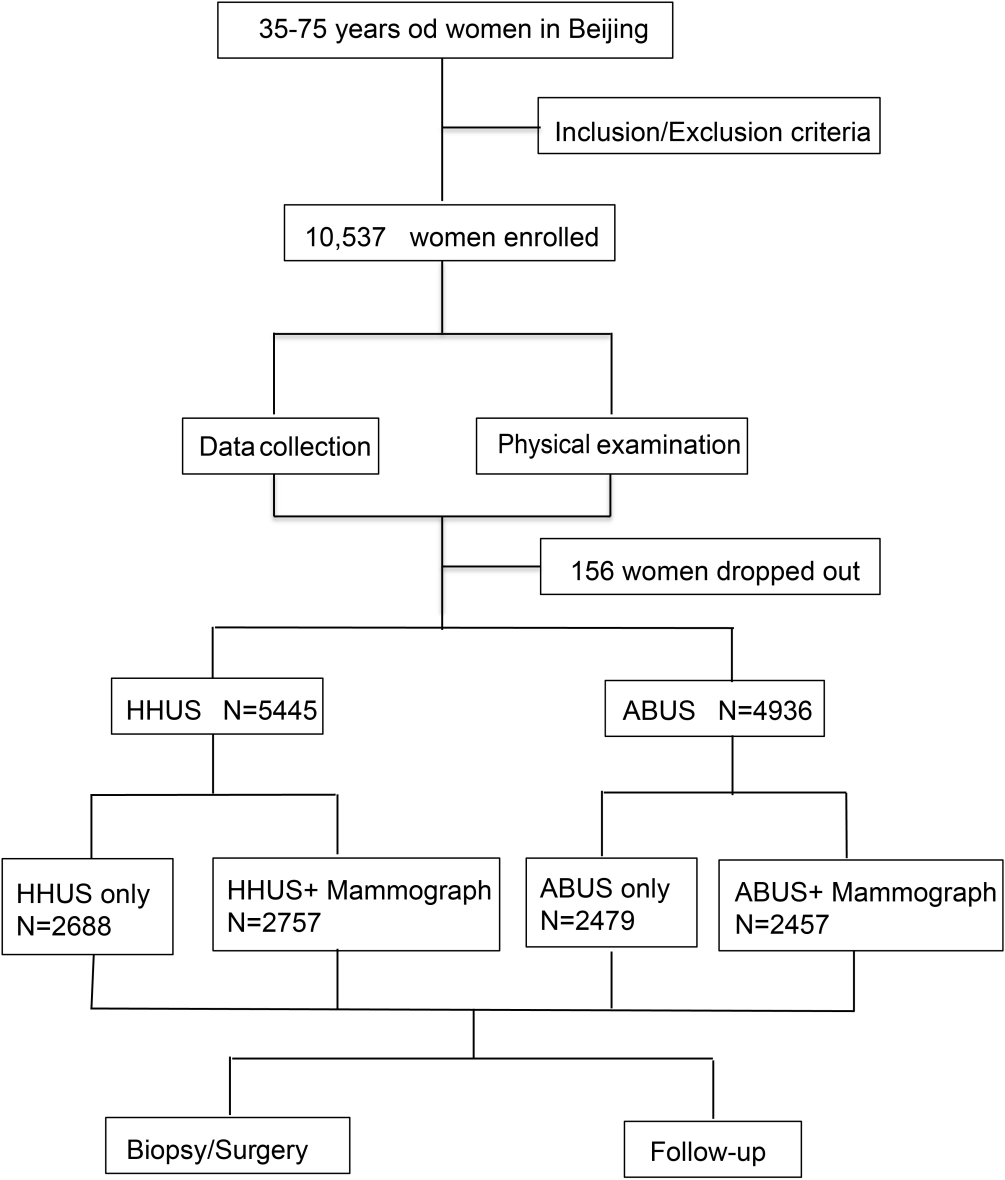
Flowchart of study design: A total of 10,537 women aged 35–75 years were enrolled in our study. After excluding 156 participants who were lost to follow-up, the number of participants enrolled in the two screening groups were 5,445 and 4,936, respectively.

This study was a multi-center randomized trial conducted in 10 breast centers across 9 districts of Beijing (Dongcheng, Chaoyang, Fengtai, Haidian, Tongzhou, Shunyi, Pinggu, Daxing, and Yanqing District). The lead center was PUMCH. The study was approved by the ethics committee of PUMCH (No. JS-1029) and registered in ClinicalTrials.gov (ChiCTR1900023916). Informed consent was obtained from all participants. Between January 1, 2016, and December 31, 2019, a total of 10,537 women aged 35–75 years from 9 districts were evaluated for study eligibility (**Figure 1**). All enrolled patients were required to fill out a high-risk factor questionnaire according to the PUMCH risk-assessment model (11). The collected data included age, height, weight, age at menarche, age at first live birth, age at menopause, breastfeeding history, lifestyle, reproductive history, and family history. Participants were randomized into two groups for HHUS or ABUS examination. Each group was further divided into subgroups based on the presence or absence of MG screening. Random number tables were used for randomization. Results of physical and ultrasound examinations, as well as follow-up outcomes, were recorded. Women with negative screening results or benign biopsy findings were invited for rescreening one year later. Medical records, telephone calls, mails, emails, or face-to-face interviews were used for annual follow-ups.

### Inclusion and exclusion criteria

Inclusion criteria: (1) Female, aged 35–75 years. (2) No history of breast cancer. (3) Willingness to provide truthful information. (4) Sign informed consent. (5) Willingness to participate in follow-up by researchers. Exclusion criteria: (1) Pregnant or breastfeeding women. (2) History of breast prosthesis implantation. (3) History of breast cancer or other malignant tumors. (4) Breast surface damage, including obvious ulcers, purulent infections, or other infections. (5) History of hysterectomy and inability to determine menstrual status.

### Screening methods and quality assurance

For HHUS screening, Resona 7 or 8 devices (Mindray Medical, Shenzhen, China) equipped with 5-14 MHz linear-array transducers were utilized. Screening with the ABUS was conducted using Siemens equipment, comprising a scanning unit and a diagnostic workstation. The scanning unit featured a high-frequency (10–15 MHz) linear transducer. All professionals involved, including physicians and technicians, underwent standardized technical, theoretical, and practical training provided by Siemens Healthcare and the Department of Ultrasound affiliated with Peking Union Medical College Hospital. Digital mammography was performed using a standard two-view technique.

Lesions were evaluated based on size, shape, border, edge, orientation, internal and posterior echo, calcification, and presence of axillary lymph nodes, among other manifestations. The Breast Imaging Reporting and Data System (BI-RADS), proposed by the American College of Radiology (ACR) in its 2013 edition, was used to classify lesions into five categories (12). Breast density was assessed via ultrasound and categorized according to BI-RADS ACR classifications and quantifications: BI-RADS A: almost entirely fat (low density of mammary gland parenchyma), BI-RADS B: scattered fibroglandular densities (average density of gland parenchyma), BI-RADS C: heterogeneously dense (high density of gland parenchyma), and BI-RADS D: extremely dense (very high density of gland parenchyma) (13).

Experienced radiologists underwent further training in ABUS and the appropriate and clear utilization of BI-RADS for quality and consistency assurance. Mammography, HHUS, and ABUS were performed and interpreted separately by different physicians with over five years of experience. Women classified as BI-RADS 4b-c or 5 underwent core needle biopsy or surgical biopsy for pathological diagnosis. For those classified as BI-RADS 3 or 4a, biopsy or follow-up was conducted as necessary. Regular annual follow-up was conducted until January 2020, with interval cancers documented during follow-up.

### Statistical analysis

Sensitivities and specificities were determined using the positive results of biopsies and follow-ups until January 2020 as the ‘gold standard’. The positive predictive value (PPV) was calculated as the malignancy rate among cases that tested positive by each screening modality. Sensitivity[True positive/(True positive+false negative)], specificity[True negative/(True negative+false positive)], accuracy[(True positive+true negative)/all], PPV(True positive/detected positive), and negative predictive value (NPV)(True negative/detected negative) for different diagnostic methods were quantified, and comparisons were made using the Chi-square test. The Receiver Operating Characteristic (ROC) curve illustrated the diagnostic performance of ABUS and HHUS, with or without mammography. A comparison of the Area Under the Curve (AUC) was conducted using the DeLong test. Statistical analyses were performed using R software (version 4.2.2). All statistical tests were two-sided, and a P-value < 0.05 was considered indicative of a significant difference.

## Results

### Participants enrollment and characteristics

A total of 10,537 women with a mean age of 47.00 ± 8.32 completed high-risk assessment and were eligible for inclusion in the study. After excluding 156 participants lost to follow-up, 10,381 participants were included in the analysis. All participants underwent a total of 35,395 breast screenings. The number of participants enrolled in the two screening groups (HHUS and ABUS) were 5,445 and 4,936, respectively. Each group was further divided into two subgroups based on the presence or absence of MG screening. The number of participants in the four subgroups (HHUS only, HHUS+MG, ABUS only, ABUS+MG) were 2,688, 2,757, 2,479, and 2,457, respectively. The demographic characteristics of the four subgroups are shown in **Supplementary Table S1**. The demographic characteristics of participants who received HHUS and ABUS are shown in **Table 1**. Generally, the demographics of the two ultrasound screening groups were comparable (P > 0.05).

**Table 1:** Demographic and clinical characteristics of enrolled patients.

Characteristics	HHUS N=5445 (%)	ABUS N=4936 (%)	*P*
Age(mean ± sd)	47.47 ± 8.16	47.47 ± 8.51	0.98
BMI(mean ± sd)	23.39 ± 2.85	23.31 ± 2.82	0.18
Menopausal status			0.07
Premenopausal	3712 (68.2)	3283 (66.5)	
Postmenopausal	1733 (31.8)	1653 (33.5)	
Times of birth			0.61
0-1	4048 (74.3)	3691 (74.8)	
≥2	1397 (25.7)	1245 (25.2)	
Times of abortion			0.48
0-1	1775 (32.6)	1641 (33.2)	
≥2	3670 (67.4)	3295 (66.8)	
Stress assessment			0.38
Low	2492 (45.8)	2278 (46.2)	
Medium	1661 (30.5)	1448 (29.3)	
High	1292 (23.7)	1210 (24.5)	
Contraceptive use or MHT			0.05
Never	4152 (76.3)	3683 (74.6)	
Past or Current	1293 (23.7)	1253 (25.4)	
Breast Volume			0.85
A-B cup	3838 (70.5)	3471 (70.3)	
C-D cup	1607 (29.5)	1465 (29.7)	
Breast Density			0.89
Fatty (<25%)	351 (6.4)	322 (6.5)	
Scattered (25-50%)	2540 (46.6)	2265 (45.9)	
Heterogeneous (51-75%)	2268 (41.7)	2083 (42.2)	
Dense (>75%)	286 (5.3)	266 (5.4)	
Family history			0.16
No	5191 (95.3)	4676 (94.7)	
Yes	254 (4.7)	260 (5.3)	
Benign breast diseases history			0.50
No	4795 (88.1)	4368 (88.5)	
Yes	650 (11.9)	568 (11.5)	

HHUS, hand-held ultrasound; ABUS, The automatic breast volume scanner ultrasound.

BMI, body mass index; MHT, Menopausal hormone therapy.

### Screening finding and Cancer detection

Ultrasound screening findings were classified according to BI-RADS score (1–3 as negative and 4–5 as positive). Among the 5,445 participants in the HHUS group, 376 subjects were diagnosed as ultrasound-positive, and 382 participants underwent biopsy or surgery. Among the 4,936 participants in the ABUS group, 350 subjects were diagnosed as ultrasound-positive, and 249 participants underwent biopsy or surgery. Overall, the HHUS group and the ABUS group identified 90 carcinomas and 292 benign lesions or 71 carcinomas and 178 benign lesions, respectively. There was no significant difference in the positive rate of ultrasound and the detection rate of carcinomas between the two groups, while the biopsy/surgery rate of the HHUS group was higher (**Table 2**). The addition of mammography to ultrasound improved the sensitivity of breast cancer screening but slightly decreased the specificity of screening (**Supplementary Table S2**).

**Table 2:** Screening findings of HHUS and ABUS.

Characteristics	HHUS N=5445 (%)	ABUS N=4936 (%)	*P*
US findings			0.71
BI-RADS0-3	5069 (93.1)	4586 (92.9)	
BI-RADS4-5	376 (6.9)	350 (7.1)	
Physical examination			<0.001
Negative	5331 (97.9)	4773 (96.7)	
Positive	114 (2.1)	163 (3.3)	
Biopsy/Operation			<0.001
No	5063 (93.0)	4687 (95.0)	
Yes	382 (7.0)	249 (5.0)	
Pathology			0.16
Benign	292 (76.4)	178 (71.5)	
Malignant	90 (23.6)	71 (28.5)	

Excluding participants without biopsy or operation.

HHUS, hand-held ultrasound; ABUS, The automatic breast volume scanner ultrasound.

The demographic characteristics of participants who underwent biopsy/surgery are shown in **Table 3**. A total of 161 malignant lesions were confirmed by pathology. Compared with 470 participants with benign lesions, patients with malignant tumors were approximately 5 years older (46.13 vs. 51.34). The proportion of postmenopausal patients was higher in patients with malignant lesions (P < 0.001). Participants with a history of benign breast disease tended to have benign lesions in this biopsy/surgery (P < 0.001). There was no significant difference in BMI, parity, abortion history, stress assessment, contraceptive use or MHT, breast volume, breast density, or family history.

**Table 3:** Demographic and clinical characteristics of participants underwent biopsy/operation.

Characteristics	Malignant N=161 (%)	Benign N=470 (%)	*P* value
Age(mean ± sd)	51.34 ± 9.35	46.13 ± 7.62	<0.001
Age			<0.001
35-44	42 (26.1)	219 (46.6)	
45-54	62 (38.5)	190 (40.4)	
55-64	42 (26.1)	52 (11.1)	
65-74	15 (9.3)	9 (1.9)	
BMI(mean ± sd)	23.30 ± 2.87	23.40 ± 2.84	0.71
Menopausal status			<0.001
Premenopausal	88 (54.7)	373 (79.4)	
Postmenopausal	73 (45.3)	97 (20.6)	
Times of birth			0.59
0-1	124 (77.0)	352 (74.9)	
≥2	37 (23.0)	118 (25.1)	
Times of abortion			0.21
0-1	59 (36.6)	147 (31.3)	
≥2	102 (63.4)	323 (68.7)	
Stress assessment			0.24
Low	57 (35.4)	202 (43.0)	
Medium	49 (30.4)	128 (27.2)	
High	55 (34.2)	140 (29.8)	
Contraceptive use or MHT			0.48
Never	115 (71.4)	349 (74.3)	
Past or Current	46 (28.6)	121 (25.7)	
Breast Volume			0.05
A-B cup	119 (73.9)	308 (65.5)	
C-D cup	42 (26.1)	162 (34.5)	
Breast Density			0.36*
Fatty (<25%)	4 (2.5)	13 (2.8)	
Scattered (25-50%)	63 (39.1)	205 (43.6)	
Heterogeneous (51-75%)	80 (49.7)	228 (48.5)	
Dense (>75%)	14 (8.7)	24 (5.1)	
Family history			0.26
No	147 (91.3)	443 (94.3)	
Yes	14 (8.7)	27 (5.7)	
Benign breast diseases history			0.006
No	136 (84.5)	492 (73.4)	
Yes	25 (15.5)	125 (26.6)	
US findings			<0.001
BIRADS0-3	68 (42.2)	368 (78.3)	
BIRADS4-5	93 (57.8)	102 (21.7)	
Physical examination			<0.001
Negative	113 (70.2)	444 (94.5)	
Positive	48 (29.8)	26 (5.5)	

*Fisher test was used for comparison.

HHUS, hand-held ultrasound; ABUS, The automatic breast volume scanner ultrasound.

BMI, body mass index; MHT, Menopausal hormone therapy.

### Diagnosis performance of HHUS and ABUS

**Figure 2:**
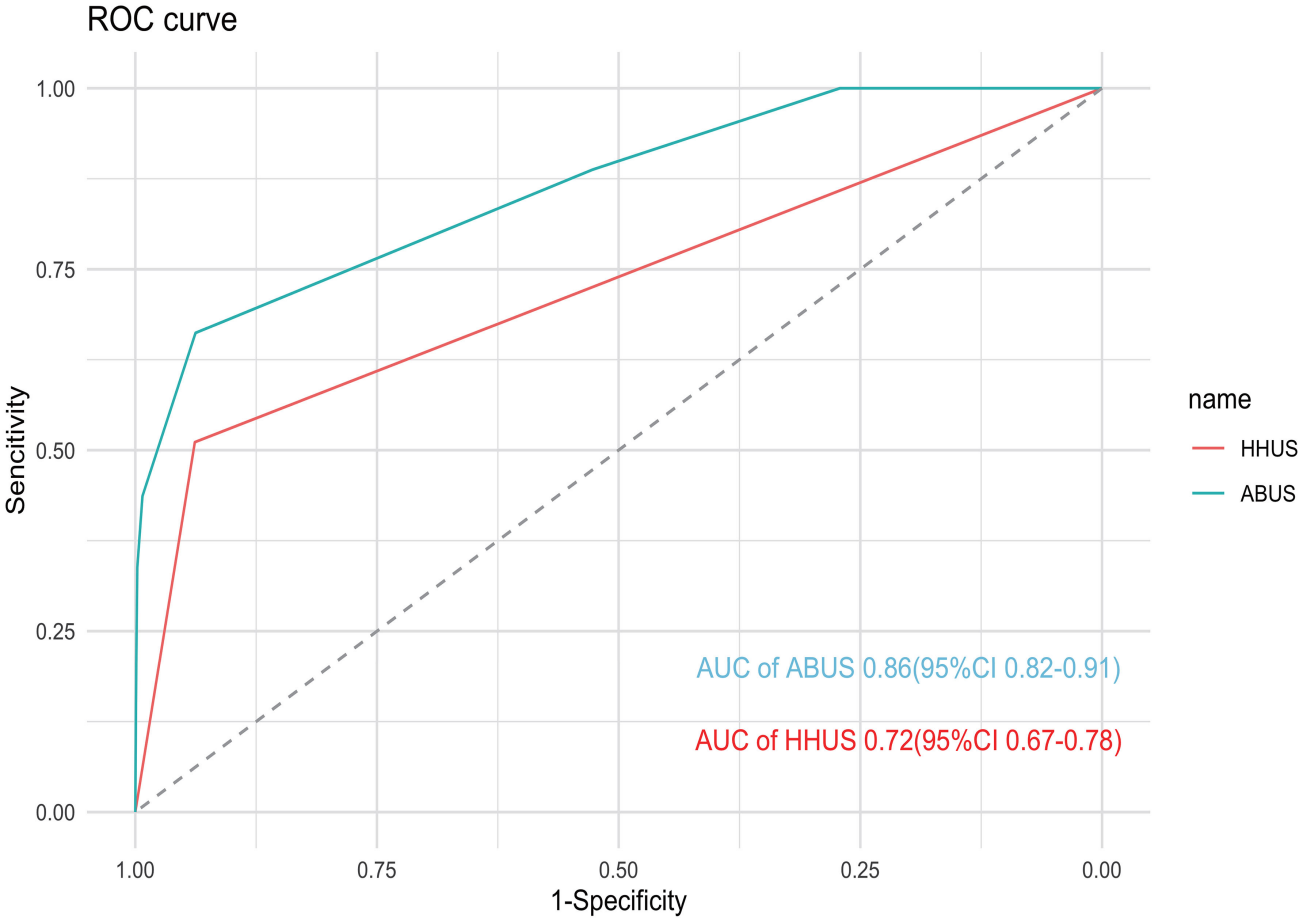
ROC curve of HHUS and ABUS for enrolled patients. The AUC of ABUS and HHUS were 0.86 (95% CI: 0.82–0.91) and 0.72 (95% CI: 0.67–0.78), respectively.

The sensitivity (SE), specificity (SP), accuracy (AC), positive predictive value (PPV), and negative predictive value (NPV) of HHUS and ABUS are calculated and presented in **Table 4**. For HHUS, the SE, SP, AC, PPV, and NPV were 51.11%, 93.84%, 93.13%, 12.23%, and 99.13%, respectively. For ABUS, these values were 66.20%, 93.77%, 93.38%, 13.43%, and 98.98%, respectively. The diagnostic performance indices between the two groups were similar (P > 0.05). ROC curves were depicted to illustrate the diagnostic performance (**Figure 2**). The AUC values of HHUS and ABUS were 0.72 (95% CI: 0.67-0.78) and 0.86 (95% CI: 0.82-0.91), respectively, indicating better diagnostic performance of ABUS compared to HHUS (Delong test, P < 0.05). The diagnostic performance indices among the four subgroups (HHUS only, HHUS+MG, ABUS only, ABUS+MG) are shown in **Supplementary Table S3**. Among the 5,214 participants who underwent mammography, the SE, SP, AC, PPV, and NPV for HHUS were 21.50%, 99.43%, 97.58%, 16.14%, and 98.37%, respectively (**Supplementary Table S4**). Compared with mammography screening, HHUS and ABUS screening demonstrated higher sensitivity in Chinese women.

**Table 4:** Diagnostic performance of HHUS and ABUS.

Rate (%)	HHUS N=5445	ABUS N=4936	X^2^	*P* value
**SE**	51.11 (46/90)	66.20 (47/71)	3.53	0.06
**SP**	93.84 (5025/5355)	93.77 (4562/4865)	2.61×10^-5^	0.99
**AC**	93.13 (5071/5445)	93.38 (4609/4936)	2.61×10^-4^	0.98
**PPV**	12.23 (46/376)	13.42 (47/350)	1.22	0.27
**NPV**	99.13 (5025/5069)	98.98(4562/4609)	1.14×10^-4^	0.99

HHUS, hand-held ultrasound; ABUS, The automatic breast volume scanner ultrasound; SE, sensitivity; SP, specificity; AC, accuracy; PPV, positive predictive value; NPV, negative predictive value.

Subgroup analyses were conducted to assess the diagnostic performance of HHUS and ABUS across patient subpopulations (**Table 5**). Participants were divided into three age subgroups: 35–44 years old (4,224 participants), 45–54 years old (4,052 participants), and 55–74 years old (2,015 participants). There was no significant difference in the diagnostic performance index between the two groups in the three different age subgroups. Similarly, the diagnostic performance index between the two groups in breast density subgroups and menopausal status subgroups was also similar (P > 0.05). Additionally, participants were divided into A-B Cup and C-D Cup subgroups based on cup size. ABUS demonstrated better sensitivity than HHUS in the C-D Cup subgroup, while there was no significant difference in SP, AC, PPV, and NPV.

**Table 5:** Subgroup analyses of diagnostic performance of HHUS and ABUS.

Subgroup/Rate		HHUS	ABUS	X ^2^	*P* value
Age					
35-44 years age group N=4224(%)	SE	44.44 (12/27)	40.00 (6/15)	2.33×10^-1^	0.63
	SP	94.07 (2047/2176)	93.57 (1877/2006)	1.33×10^-3^	0.97
	AC	93.46 (2059/2203)	93.17 (1883/2021)	4.51×10^-4^	0.98
	PPV	8.51 (12/141)	4.44 (6/135)	1.28	0.26
	NPV	99.27 (2047/2062)	99.52 (1877/1886)	3.14×10^-4^	0.99
45-54 years age group N=4052	SE	54.55 (18/33)	72.41 (21/29)	2.51	0.11
	SP	93.79 (1979/2110)	93.67 (1761/1880)	7.68×10^-5^	0.99
	AC	93.19 (1997/2143)	93.35 (1782/1909)	1.37×10^-4^	0.99
	PPV	12.08 (18/149)	15.00 (21/140)	3.15×10^-1^	0.57
	NPV	99.25 (1979/1994)	99.55 (1761/1769)	4.53×10^-4^	0.98
55-74 years age group N=2105	SE	53.33 (16/30)	74.07 (20/27)	3.38	0.07
	SP	93.45 (999/1069)	94.38 (924/979)	4.60×10^-3^	0.95
	AC	92.36 (1015/1099)	93.84 (944/1006)	1.18×10^-2^	0.91
	PPV	18.60 (16/86)	26.67 (20/75)	1.44	0.23
	NPV	98.62 (999/1013)	99.25 (924/931)	2.01×10^-3^	0.96
Breast Density					
Fatty (<25%) + Scattered (25-50%) N=5478	SE	47.73 (21/44)	56.52 (13/23)	7.41×10^-1^	0.39
	SP	95.79 (2727/2847)	95.44 (2447/2564)	6.41×10^-4^	0.98
	AC	95.05 (2748/2891)	95.09 (2460/2587)	8.41×10^-6^	0.99
	PPV	14.89 (21/141)	10.00 (13/130)	9.61×10^-1^	0.33
	NPV	99.16 (2727/2750)	99.59 (2447/2457)	9.30×10^-4^	0.98
Heterogeneous (51-75%) + Dense (>75%) N=4903	SE	54.35 (25/46)	70.83 (34/48)	2.17	0.14
	SP	91.63 (2298/2508)	91.92 (2115/2301)	4.58×10^-4^	0.98
	AC	90.96 (2323/2554)	91.49 (2149/2349)	1.53×10^-3^	0.97
	PPV	10.64 (25/235)	15.45 (34/220)	8.87×10^-1^	0.35
	NPV	99.09 (2298/2319)	99.34 (2115/2129)	3.15×10^-4^	0.99
Breast Volume					
A-B cup N=7309	SE	57.35 (39/68)	62.75 (32/51)	2.43×10^-1^	0.62
	SP	94.08 (3547/3770)	93.95 (3213/3420)	8.99×10^-5^	0.99
	AC	93.43 (3586/3838)	93.49 (3245/3471)	1.93×10^-5^	0.99
	PPV	14.89 (39/262)	13.39 (32/239)	7.96×10^-2^	0.78
	NPV	99.19 (3547/3576)	99.41 (3213/3232)	2.44×10^-4^	0.99
C-D cup N=3072	SE	31.82 (7/22)	75.00 (15/20)	17.46	**<0.001**
	SP	93.25 (1478/1585)	93.36 (1349/1445)	6.48×10^-5^	0.99
	AC	92.41 (1485/1607)	93.11 (1364/1465)	2.64×10^-3^	0.96
	PPV	6.14 (7/114)	13.51 (15/111)	2.76	0.10
	NPV	98.99 (1478/1493)	99.63 (1349/1354)	2.06×10^-3^	0.96
Menopausal status					
Premenopausal N=6995	SE	50.00 (27/54)	58.82 (20/34)	7.15×10^-1^	0.40
	SP	93.90 (3435/3658)	93.07 (3024/3249)	3.68×10^-3^	0.95
	AC	93.27 (3462/3712)	92.72 (3044/3283)	1.63×10^-3^	0.97
	PPV	10.80 (27/250)	8.16 (20/245)	3.68×10^-1^	0.54
	NPV	99.22 (3435/3462)	99.54 (3024/3038)	5.15×10^-4^	0.98
Postmenopausal N=3386	SE	52.78 (19/36)	72.97 (27/37)	3.24	0.07
	SP	93.69 (1590/1697)	95.17 (1538/1616)	1.16×10^-2^	0.91
	AC	92.84 (1609/1733)	94.68 (1565/1653)	1.81×10^-2^	0.89
	PPV	15.08 (19/126)	25.71 (27/105)	2.77	0.10
	NPV	98.94 (1590/1607)	99.35 (1538/1548)	8.48×10^-4^	0.98

HHUS, hand-held ultrasound; ABUS, The automatic breast volume scanner ultrasound; SE, sensitivity; SP, specificity; AC, accuracy; PPV, positive predictive value; NPV, negative predictive value.

## Discussion

Early detection of breast cancer has significantly reduced breast cancer mortality by 15% and led to down-staging of breast cancer at diagnosis (14). While MG is the preferred screening method in Western countries, previous research has shown that ultrasound examination is sensitive, specific, and cost-effective for detecting breast cancer in Chinese women (9). In our study, ultrasound screening demonstrated higher sensitivity compared to MG. HHUS is operator-dependent and has poor repeatability, whereas ABUS overcomes the limitations of manual scanning and offers repeatability. In 2012, ABUS was approved for use in the United States and subsequently introduced to China. It stands as the sole ultrasound system globally to have obtained FDA certification for the early diagnosis of breast cancer. ABUS offers standardized tomographic images of the breast that have already found applications in clinical practice. ABUS has emerged as a novel breast cancer diagnostic technology and shows no statistically significant difference from HHUS in terms of interobserver variability and diagnostic performance (15). In this multi-center randomized trial, ABUS was used as a diagnostic tool for the first time in large-scale population opportunistic screening, and its diagnostic performance was similar to HHUS (P > 0.05). Combining ultrasound with mammography in our study improved screening sensitivity but slightly reduced specificity, though the difference in specificity was not significant. Previous studies have demonstrated that ABUS has better or at least comparable diagnostic capabilities to HHUS. For instance, in a study with over 400 lesions, ABUS showed a sensitivity and specificity of 92.16% and 87.05%, respectively, while HHUS demonstrated a sensitivity and specificity of 90.20% and 84.17%, respectively, with no significant difference from ABUS (16). Another study conducted in Beijing reported a sensitivity of 91.8% and 94.7% and a specificity of 92.9% and 89.4% for ABUS and HHUS, respectively, with similar diagnostic accuracy. The authors attributed these findings to ABUS’s ability to display more coronal plane-related information, such as mass margins, shape, spiculations, and tissue retraction distortion (17). A meta-analysis of 14 studies showed that ABUS has a sensitivity range of 0.72–1.0 and a specificity range of 0.52–0.98, while HHUS has a sensitivity range of 0.62–1.0 and a specificity range of 0.49–0.99 (10). In summary, ABUS has demonstrated consistent sensitivity and specificity in our study and previous research, indicating its utility for breast disease diagnosis and breast cancer screening.

Another advantage of ABUS in breast cancer screening its potential for teleconsultation compared to HHUS. ABUS images can be stored for diagnosis and consultation by different radiologists, addressing issues of result deviation due to variations in radiologists’ experience in breast screening. In our study, ABUS demonstrated higher sensitivity than HHUS in breast cancer screening, with no compromise in specificity, indicating superior diagnostic performance. Subgroup analysis revealed no significant differences in diagnostic indicators between the two ultrasound examinations across age and breast density subgroups, consistent with findings from Xin’s research (17). Xin’s study showed that ABUS sensitivity and specificity are comparable to HHUS across different age and breast density subgroups. In different breast volume subgroups, ABUS exhibited higher sensitivity in the C-D cup group. This could be attributed to manual ultrasound potentially missing areas in larger breasts, while ABUS, with its machine scanning, does not encounter such issues. ABUS is more advantageous in screening larger breasts compared to smaller ones. Although the AUC of mammograms for non-dense breasts is significantly better than that for dense breasts (18), the similar imaging principles of both types of ultrasound result in no significant difference in sensitivity and specificity between HHUS and ABUS across different breast density subgroups. Patients undergoing screening ultrasound typically have higher breast density, younger age, and potentially higher breast cancer risk compared to those with non-dense breasts (3). Mammographic breast density exhibits a significant negative correlation with age (19), and there is no significant difference in the utilization of the two types of ultrasound between premenopausal and postmenopausal women.

From a health economics perspective, ABUS can significantly reduce labor costs, provided there is no significant difference in diagnostic ability between the two types of ultrasound. ABUS requires lower experience and technical expertise from operators, thus reducing training costs for screening physicians. Moreover, with machine learning, complete ABUS imaging records can be diagnosed and analyzed with the assistance of artificial intelligence, further lowering labor costs (20).

This study has several limitations. Firstly, despite randomizing screened patients into ABUS and HHUS groups, there may still be some selection bias, although no significant differences in baseline data were observed between the two groups. Additionally, due to the less clinical use of ABUS compared to HHUS, some participants assigned to the ABUS group declined examination and follow-up, resulting in a slightly lower number of participants in the ABUS group. Secondly, mortality was not assessed in this study; instead, the detection rate was used as an alternative endpoint. Thirdly, the follow-up time in this study is relatively short, which precludes reporting on mortality rate. Repeated screening or extended follow-up could provide more informative data for breast cancer screening.

In summary, data from this multi-center study suggest that ABUS could be more sensitive and potentially superior to HHUS in breast cancer screening. ABUS is user-friendly, requires less training, and reduces the reliance on examiner experience compared to traditional ultrasound. The integration of automatic ultrasound with artificial intelligence holds promising prospects for future large-scale breast cancer screening efforts.

## Data Availability

The raw data supporting the conclusions of this article will be made available by the authors, without undue reservation.
