# Supplementary material for: A multi-centre, randomised trial for diagnostic efficacy of the automatic breast volume scanner ultrasound for breast cancer screening in China
Source: Front Oncol. 2025 Jan 21;14:1421425. doi: 10.3389/fonc.2024.1421425 (PMC11790623; doi:10.3389/fonc.2024.1421425)
Supplement: Supplementary file 1 [file Table1.docx]

Supplementary Materials

Table S1. Demographic and clinical characteristics of enrolled Patients according to four groups.

| Characteristics | HHUS only  N=2688(%) | HHUS+MG  N=2757(%) | ABUS only  N=2479 (%) | ABUS+MG  N=2457(%) | *P* value |
| --- | --- | --- | --- | --- | --- |
| Age(mean±sd) | 46.65±8.34 | 48.27±7.89 | 46.17±8.56 | 48.78±8.26 | <0.001 |
| BMI(mean±sd) | 23.28±2.84 | 23.50±2.86 | 23.23±2.86 | 23.40±2.78 | 0.009 |
| Menopausal status |  |  |  |  | <0.001 |
| Premenopausal | 1892(70.4) | 1820(66.0) | 1765(71.2) | 1518(61.8) |  |
| Postmenopausal | 796(29.6) | 937(34.0) | 714(28.8) | 939(38.2) |  |
| Times of birth |  |  |  |  | 0.66 |
| 0-1 | 2008(74.7) | 2040(74.0) | 1869(75.4) | 1822(74.2) |  |
| ≥2 | 680(25.3) | 717(26.0) | 610(24.6) | 635(25.8) |  |
| Times of abortion |  |  |  |  | 0.34 |
| 0 | 884(32.9) | 891(32.3) | 851(34.3) | 790(32.2) |  |
| ≥1 | 1803(67.1) | 1866(67.7) | 1682(65.7) | 1667(67.8) |  |
| Stress assessment |  |  |  |  | 0.22 |
| Low | 1228(45.7) | 1264(45.8) | 1103(44.5) | 1175(47.8) |  |
| Medium | 829(30.8) | 832(30.2) | 759(30.6) | 689(28.0) |  |
| High | 631(23.5) | 661(24.0) | 617(24.9) | 593(24.1) |  |
| Contraceptive use or MHT |  |  |  |  | 0.11 |
| Never | 2028(75.4) | 2124(77.0) | 1840(74.2) | 1843(75.0) |  |
| Past or Current | 660(24.6) | 633(23.0) | 639(25.8) | 614(25.0) |  |
| Breast Volume |  |  |  |  | 0.003 |
| A-B cup | 1956(72.8) | 1882(68.3) | 1757(70.9) | 1714(69.8) |  |
| C-D cup | 732(27.2) | 875(31.7) | 722(29.1) | 743(30.2) |  |
| Breast Density |  |  |  |  | 0.37 |
| Fatty (<25%) | 194(7.2) | 157(5.7) | 158(6.4) | 164(6.7) |  |
| Scattered (25-50%) | 1252(46.6) | 1288(46.7) | 1114(44.9) | 1151(46.8) |  |
| Heterogeneous (51-75%) | 1096(40.8) | 1172(42.5) | 1076(43.4) | 1007(41.0) |  |
| Dense (>75%) | 146(5.4) | 131(5.3) | 140(5.1) | 13.5(5.5) |  |
| Family history |  |  |  |  | 0.02 |
| No | 2582(96.1) | 2609(94.6) | 2359(95.2) | 2317(94.3) |  |
| Yes | 106(20.6) | 148(28.8) | 120(23.3) | 140(27.2) |  |
| Benign breast diseases history |  |  |  |  | <0.001 |
| No | 2388(88.8) | 2407(87.3) | 2237(90.2) | 2131(86.7) |  |
| Yes | 300(11.2) | 350(12.7) | 242(9.8) | 326(13.3) |  |

HHUS:hand-held ultrasound; ABUS:The automatic breast volume scanner ultrasound; MG:mammograph; BMI: body mass index; MHT: Menopausal hormone therapy.

Table S2. Screening findings of four screening groups

|  | HHUS only  N=2688(%) | HHUS+MG  N=2757(%) | ABUS only  N=2479 (%) | ABUS+MG  N=2457(%) | *P* value |
| --- | --- | --- | --- | --- | --- |
| Screening findings |  |  |  |  | <0.001 |
| BI-RADS0-3 | 2557(95.1) | 2512(91.1) | 2363(95.3) | 2223(90.5) |  |
| BI-RADS4-5 | 131(4.9) | 245(8.9) | 116(4.7) | 234(9.5) |  |
| Physical examination |  |  |  |  | <0.001 |
| Negative | 2640(98.2) | 2691(97.6) | 2429(98.0) | 2344(95.4) |  |
| Positive | 48(1.8) | 66(2.4) | 50(2.0) | 113(4.6) |  |
| Biopsy/Operation |  |  |  |  | <0.001 |
| No | 2556(95.1) | 2507(90.9) | 2398(96.7) | 2289(93.2) |  |
| Yes | 132(4.9) | 250(9.1) | 81(3.3) | 168(6.8) |  |
| Pathology of biopsy/operation^#^ |  |  |  |  | 0.14 |
| Benign | 105(79.5) | 187(74.8) | 63(77.8) | 115(68.5) |  |
| Malignant | 27(20.5) | 63(25.2) | 18(22.2) | 53(31.5) |  |

^#^ Excluding participants without biopsy or operation.

HHUS:hand-held ultrasound; ABUS:The automatic breast volume scanner ultrasound; MG:mammograph;

Table S3. Diagnostic performance of HHUS only, HHUS+MG, ABUS only and ABUS+MG

|  | HHUS only  N=2688(%) | HHUS+MG  N=2757(%) | ABUS only  N=2479 (%) | ABUS+MG  N=2457(%) | X ^2^ | *P* value |
| --- | --- | --- | --- | --- | --- | --- |
| SE | 37.04(10/27) | 57.14(36/63) | 44.44(8/18) | 73.58(39/53) | 14.49 | **0.002** |
| SP | 95.45(2540/2661) | 92.24(2485/2694) | 95.61(2353/2461) | 91.89(2209/2404) | 0.129 | 0.988 |
| AC | 94.87(2550/2688) | 91.44(2521/2757) | 95.24(2361/2479) | 91.49(2248/2457) | 0.139 | 0.987 |
| PPV | 7.63(10/131) | 14.69(36/245) | 6.90(8/116) | 16.67(39/234) | 6.366 | 0.095 |
| NPV | 99.34(2540/2557) | 98.93(2485/2512) | 99.58(2353/2363) | 99.37(2209/2223) | 0.002 | 0.999 |

HHUS:hand-held ultrasound; ABUS:The automatic breast volume scanner ultrasound; MG:mammograph;SE: sensitivity; SP: specificity; AC: accuracy; PPV: positive predictive value; NPV: negative predictive value.

Table S4. Diagnostic performance of HHUS, ABUS and MG

| Rate (%) | HHUS  N=5445 | ABVS  N=4936 | MG  N=5214 | X^2^ | *P* value |
| --- | --- | --- | --- | --- | --- |
| SE | 51.11(46/90) | 66.20(47/71) | 21.50(23/107) | 22.35 | <0.001 |
| SP | 93.84(5025/5355) | 93.77(4562/4865) | 99.43(5065/5094) | 0.22 | 0.896 |
| AC | 93.13(5071/5445) | 93.38(4609/4936) | 97.58(5088/5214) | 0.13 | 0.936 |
| PPV | 12.23(46/376) | 13.42(47/350) | 16.14(61/378) | 1.88 | 0.389 |
| NPV | 99.13(5025/5069) | 98.98(4562/4609) | 98.37(5065/5149) | 0.003 | 0.998 |

HHUS:hand-held ultrasound; ABUS:The automatic breast volume scanner ultrasound; MG:mammograph; SE: sensitivity; SP: specificity; AC: accuracy; PPV: positive predictive value; NPV: negative predictive value.
